# A systematic development process for patient decision aids

**DOI:** 10.1186/1472-6947-13-S2-S2

**Published:** 2013-11-29

**Authors:** Angela Coulter, Diana Stilwell, Jennifer Kryworuchko, Patricia Dolan Mullen, Chirk Jenn Ng, Trudy van der Weijden

**Affiliations:** 1 Department of Public Health, University of Oxford, Rosemary Rue Building, Old Road Campus, Headington, Oxford, OX3 7LF, UK; 2 Informed Medical Decisions Foundation, 40 Court Street, Suite 300, Boston, MA 02108, USA; 3 College of Nursing, University of Saskatchewan, St. Andrew’s College, 312, 1121 College Drive, Saskatoon, SK Canada S7N 0W3; 4 University of Texas Health Science Center, Division of Health Promotion & Behavioral Sciences, University of Texas School of Public Health, 7000 Fannin St., UCT Ste 2522, Houston, Texas 77030, USA; 5 Department of Primary Care Medicine, Faculty of Medicine, University of Malaya, 50603 Kuala Lumpur, Malaysia; 6 Dept of General Practice, CAPHRI School for Public Health and Primary Care, Maastricht University PO Box 616, 6200 MD Maastricht, the Netherlands

## Abstract

**Background:**

The original version of the International Patient Decision Aid Standards (IPDAS) recommended that patient decision aids (PtDAs) should be carefully developed, user-tested and open to scrutiny, with a well-documented and systematically applied development process. We carried out a review to check the relevance and scope of this quality dimension and, if necessary, to update it.

**Methods:**

Our review drew on three sources: a) published papers describing PtDAs evaluated in randomised controlled trials and included in the most recent Cochrane Collaboration review; b) linked papers cited in the trial reports that described how the PtDAs had been developed; and c) papers and web reports outlining the development process used by organisations experienced in developing multiple PtDAs. We then developed an extended model of the development process indicating the various steps on which documentation is required, as well as a checklist to assess the frequency with which each of the elements was publicly reported.

**Results:**

Key features common to all patient decision aid (PtDA) development processes include: scoping and design; development of a prototype; ‘alpha’ testing with patients and clinicians in an iterative process; ‘beta’ testing in ‘real life’ conditions (field tests); and production of a final version for use and/or further evaluation. Only about half of the published reports on the development of PtDAs that we reviewed appear to have been field tested with patients, and even fewer had been reviewed or tested by clinicians not involved in the development process. Very few described a distribution strategy, and surprisingly few (17%) described a method for reviewing and synthesizing the clinical evidence. We describe a model development process that includes all the original elements of the original IPDAS criterion, expanded to include consideration of format and distribution plans as well as prototype development.

**Conclusions:**

The case for including each of the elements outlined in our model development process is pragmatic rather than evidence-based. Optimal methods for ensuring that each stage of the process is carried out effectively require further development and testing.

## Background

The need for a systematic and transparent process for developing patient decision aids (PtDAs) was recognised in the original version of the International Patient Decision Aid Standards (IPDAS) [1]. Specific developmental steps described in the first iteration of IPDAS were: a) assessing decisional needs, including analysis of the characteristics of the decision, specification of treatment options, outcomes and probabilities, and of patients’ information needs and their requirements for decisional support; b) formation and composition of groups to develop and review decision aids; c) methods for drafting, reviewing and revising these; d) field testing with patients; and e) external peer review or critical appraisal by people not involved in its development.

These steps were originally identified through scrutiny of 55 reports of trials that were included in the second update of a Cochrane Collaboration review on the effectiveness of patient decision aids [2], together with accompanying papers on how these had been developed. However, the field has moved on since then; many more PtDAs have been developed and evaluated, including web-based tools, the Cochrane Collaboration review has been updated and now includes 86 randomised controlled trials [3], and several more groups have published details of their PtDA development process. We therefore decided to repeat and update the review to check the currency and relevance of the original definition and scope of this quality standard.

## Theoretical rationale for evaluating patient decision aids on this quality standard

It is important that PtDAs are carefully developed, user-tested, and open to scrutiny, with a well-documented and systematically applied development process. Some decision aids have been designed for one-off use in studies to advance knowledge, while others are intended for wider use in a range of real-life clinical settings. Some have been developed by academics, some by clinicians, some by voluntary organizations, and some by commercial companies. Some developers have produced only one or two PtDAs, while others have designed processes for producing them at scale. Whatever their provenance or purpose, users (clinicians and patients) require assurance that the development process has been carried out to acceptable standards. Poor quality decision aids have the potential to cause harm and they are less likely to advance implementation of shared decision making more broadly, so it is essential that users are provided with sufficient documentation to check the validity and reliability of the development processes.

Next to validity and reliability, it is also considered important to involve a range of stakeholders in the development process. As well as playing a key role in design of the PtDA, the involvement of patients, clinicians, and other relevant experts—for example patient educators, people with specific expertise in shared decision making, or policy makers—can facilitate successful implementation by addressing barriers to delivering or using the PtDA. Clinicians who do not trust or agree with the content of a PtDA are unlikely to encourage their patients to view it [4].

## Empirical evidence

### Review methods

Our review focused on descriptions of the PtDA development process. We obtained these descriptions from three main sources: a) published papers describing 86 PtDAs that had been evaluated in randomised controlled trials and included in the latest update of the Cochrane Collaboration review of decision aids [3]; b) linked papers cited in the trial reports that provided expanded descriptions of how the PtDAs had been developed; and c) papers and web reports describing the development process used by a convenience sample of organisations that had developed several decision aids and had published papers or web reports describing their development process. The checklist we used to review these descriptions of the PtDA development process appears in Additional File 1, Appendix 1.

The PtDA development process has been described in a number of trial reports and associated articles. Many of the studies included in the Cochrane Collaboration review gave only cursory descriptions of how their PtDAs had been developed, but several linked papers provided useful supplementary detail [5-16] . Many PtDAs reported in the trials included in the Cochrane Collaboration review were ‘one-offs’ designed for research purposes rather than for wider distribution, and therefore did not generate insights into the implementation issues that might have been addressed during development. However, the experience of organisations involved in developing series of PtDAs is helpful for fleshing out the details of what is involved. Some groups that have developed multiple PtDAs have proposed guidelines for their development [17-20], or described insights generated by a particular approach [21]. The following is a brief overview of selected approaches to decision aid development.

### Processes used by experienced PtDA developers

#### Ottawa Decision Support Framework

O’Connor is among the earliest authors to describe the development of a PtDA, and the Ottawa Decision Support Framework (ODSF) guided the development of at least 22 of the PtDAs included in the Cochrane Collaboration review [10,18]. Based on expectancy value, decisional conflict, and social support theories, the framework includes three key elements: 1) assessment of determinants of decisions (both patients’ and providers’); 2) provision of decision support interventions to prepare the patient and provider to make and implement a decision; and 3) evaluation of the success of the interventions at improving the quality and outcomes of the decision process. Additional detail is provided to define determinants of decisions, such as socio-demographic and clinical characteristics; patients’ and providers’ perceptions of the decision and of what important others think about the decision; and resources (both personal and external) available to make the decision.

The authors noted that the goals of decision support are to address modifiable and suboptimal decision determinants, such as inadequate knowledge, unrealistic expectations, unwanted pressure, and inadequate support. They encouraged the use of tailored outcome probabilities, detailed descriptions of benefits and risks, and information on the opinions and perspectives of others (both clinicians and patients) on the decision. Using the example of a decision aid aimed at helping women decide about use of postmenopausal hormone therapy, O’Connor outlined an iterative development process involving the research team and panels of patients and experts, with the PtDA content based on clinical guidelines, and structured guidance in clarifying values and implementing a decision is provided by a personal worksheet [18].

The Ottawa Framework is particularly relevant to ‘preference-sensitive’ decisions, which involve careful deliberation and consideration of trade-offs among options. Perhaps because the PtDA developed by O’Connor was based on an existing high-quality clinical guideline, the framework provides little advice for how developers should review and synthesize the relevant clinical evidence. The framework also does not address how developers might deal with conflicts of interest, achieving consensus on the evidence, maintaining the PtDA content over time, or implementation outside research settings.

#### Cardiff University

Based on their experience of developing three web-based PtDAs over a seven-year period, Elwyn and colleagues proposed a development process for web-based decision support interventions [17]. This systematic ‘process map’ includes three main steps: 1) content specification, with an emphasis on ensuring that patients’ perspectives on the proposed options are sought and included in addition to synthesis of the scientific evidence; 2) design, including storyboarding, an iterative phase of trial and experimentation called “sandpit” testing, and usability testing; and 3) field testing with patients facing the decision and clinicians who are interacting with them. The process calls for extensive documentation, including: a protocol document that explains the decision and highlights the rationale for developing a PtDA; evidence synthesis based on systematic reviews or comprehensive literature searches; storyboard; and technical specification document to guide the website development.

The Cardiff projects were overseen by a project management group, which retained editorial control, and included involvement at all steps by key stakeholders including clinicians, patients, and policymakers. Unique challenges faced by developers of web-based tools are highlighted, including decisions regarding navigation (free versus mandated) and use of interactivity (audio, video, gaming, avatars, etc.) in ways that add value and enhance ease of use yet avoid over-engineering. The authors found little evidence to inform best practices in these areas.

The process outlined by Elwyn and colleagues is widely applicable across a range of situations for which decision support interventions may be developed (i.e., screening, treatment, etc.) and a variety of media (although some of the concepts included, such as storyboarding, are adapted from film production). However, as the authors acknowledge, the process is time-consuming and costly: three PtDAs developed using this process each took two to three years to develop and test. Insights gained from early efforts could be generalized to create templates to allow more efficient, less costly future development. The process outlined does not offer recommendations regarding conflict of interest, processes for achieving consensus on the evidence, maintenance of PtDA content over time, or implementation outside research settings.

#### Dutch Institute for Healthcare Improvement

Researchers at the Dutch Institute for Healthcare Improvement reported on the development over a 12-month period of 6 decision aids based on existing evidence-based clinical guidelines [19]. Citing the Ottawa Decision Support Framework and the IPDAS standards, the authors followed four key steps: 1) establishment of criteria and selection of topics; 2) assessment of patients’ information needs via literature review and focus groups; 3) drafting of the aid, including iterative review by a multidisciplinary working group of health professionals, guideline developers, decision-making experts, and patients, and with reference to existing aids on the topic; and 4) endorsement of the aid and establishment of ownership and responsibility for the maintenance and updating of both the supporting guideline and the decision aid itself. With regard to implementation, the authors call for concomitant development and coordinated release of clinical practice guidelines and accompanying PtDAs that support their application, and for identifying and acknowledging early in the guideline development process any so-called ‘grey zones’ of uncertainty regarding patient preferences.

The outlined process appears efficient and scalable when high-quality evidence-based practice guidelines are available. However, the authors acknowledge that additional research is needed to evaluate the effect of the aids in practice within the Dutch health care system. The evidence synthesis step used by other developers is addressed by use of national evidence-based guidelines; the authors note that these should also meet internationally accepted quality criteria, for example the AGREE guidance [22]. The resulting tools include a values-clarification method, but the process by which the method was selected and ‘populated’ with non-directive, standardized questions is not defined.

#### Mayo Clinic

Montori and colleagues described insights gleaned from the pragmatic process they followed to develop the Statin Choice decision aid for patients with diabetes, which was evaluated in an RCT included in the Cochrane Collaboration review [21]. In particular, the authors describe how observations of clinical interactions during office visits, and of early prototypes in use during patient-provider encounters, can inform the ultimate format, design, and content of the final PtDA. Similar to other developers, their experience reinforces the importance of flexibility, iteration, and involvement of patients and clinicians throughout the process. This article does not recommend a particular development process, but rather offers insights unique to the approach their research group chose. The direct observation methodology that Montori and colleagues describe may complement more traditional needs assessment approaches for informing developers about what patients and physicians need from a PtDA. Observing early prototypes in the setting they are being designed for can also be an important step in ensuring that the intervention will work as intended and have the desired effect on the decision making process. These insights also highlight the importance of flexibility during the early stages of design and development.

#### Informed Medical Decisions Foundation

Developers that produce PtDAs for both research purposes and for public distribution, such as the Informed Medical Decisions Foundation (IMDF), provide details of their development process on their organization’s website (http://www.informedmedicaldecisions.org). Ten of the RCTs included in the most recent update of the Cochrane review used PtDAs developed by this process. IMDF lists the following elements in their PtDA development process: 1) involvement of healthcare providers representing key clinical specialties, overseen by a clinician who divests him or herself of any potential financial conflicts of interest; 2) involvement of patients at several stages, including needs assessment via focus groups and literature reviews; and 3) review and evaluation of PtDA drafts by providers and patients prior to their release for general use. The approach outlines processes for evidence review and synthesis, disclosure of funding source and conflicts of interest, and periodic review and updates.

## A model development process

**Figure 1:**
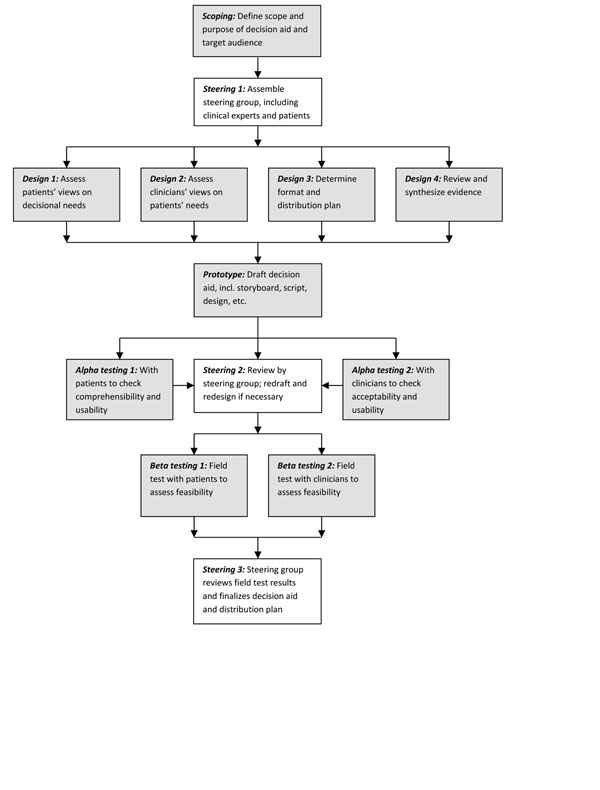
Model Development Process for Decision Aids

Figure 1 illustrates our group’s consensus on the main elements of a systematic development process based on our review of relevant literature. While different authors have placed greater or lesser emphasis on particular aspects, key features common to all include scoping and design, development of a prototype, ‘alpha’ testing with patients and clinicians in an iterative process (testing by people directly involved in the development process), ‘beta’ testing in ‘real life’ conditions (field tests with patients and clinicians not involved in the development process), and production of a final version for use and/or further evaluation. The process is often overseen by a multidisciplinary steering group that includes patient and clinician representatives and other relevant stakeholders. The specific details of how the prototype is developed, in particular how material for inclusion is reviewed and selected, are important elements that shape the final product. Less often mentioned explicitly are choice of format and considerations of how the PtDA will be distributed and made available to patients and clinicians, yet this seemed to us to be a crucial part of a design process for a tool intended for clinical practice.

Further details from our review of the reports included in the Cochrane Collaboration systematic review are included in Additional File 2, Table S1, together with details of the frequency at which the key elements in our model were reported.

As far as we could tell, few PtDAs evaluated in the Cochrane Collaboration review had undergone all the steps outlined in our model during their development. Only about half appear to have been field tested with patients, and even fewer had been reviewed or tested by clinicians not involved in the development process. Inclusion in the Cochrane Collaboration review meant that all the PtDAs had been evaluated in randomised controlled trials, but this type of post-hoc evaluation of impact serves a different purpose from that of alpha and beta testing during the development process. Very few developers described a distribution strategy, and surprisingly few (17%) described a method for reviewing and synthesizing the clinical evidence. In many cases it was difficult to gauge from the trial reports whether a development process along the lines recommended in IPDAS had been followed or not.

## Discussion

Despite the proliferation of patient decision aids, detailed information on the processes by which they were developed is limited. It was disappointing to find that many of the PtDA trial reports failed to provide clear information about how their tools were developed, but it is important to bear in mind that most of these were developed before the publication of the IPDAS criteria. A recent study showed that PtDAs that scored highly on having a systematic development process also achieved high scores against other IPDAS criteria [23], but we found no hard evidence to support the hypothesis that a systematic development process results in a demonstrably better gain when using PtDAs in randomised trial or “real life” conditions. Nevertheless, we believe the case for adopting a systematic and transparent process is well made.

Our new, more comprehensive model of the development process includes all the elements of the original (see ‘Background’ above), but they have been renamed and reordered to clarify the different phases of the development process. The description of PtDA design has been expanded to include consideration of format and distribution plans, and we have also added a section on prototype development. Our aim was to provide a clearer overview of the entire development process. However, this is an overview only, and much uncertainty remains about the best way to tackle many of the individual elements. There is little evidence on the relative importance of each of the features; most have emerged from practical experience supported by consensus, but we do not claim they are evidence-based.

Optimal methods for determining patients’ decisional needs require further development and testing. Studies should evaluate how much information patients want or need and how much detail is required. There is currently no consensus on how to select material for inclusion in decision aids, yet the selection process is crucial and frequently debated among those involved. The recent development of short-form PtDAs for use within consultations offers an opportunity to compare the effects of brief PtDAs against those that are lengthier and more detailed [24]. There may be scope for closer alignment between the development of clinical guidelines and PtDAs since they draw on the same evidence base. This might lead to greater efficiency in the development process and better acceptance of PtDAs by clinicians [25].

More guidance is needed to inform PtDA alpha- and beta- tests, including user-centred design methods, acceptability, usability, and feasibility testing. While it is probably unreasonable to expect every PtDA to be evaluated in an RCT, we believe that all PtDAs should be subject to user testing at some stage during their development. For those developing PtDAs at scale, it may not be necessary to conduct beta (usability) testing for each new product once they have designed and tested in a process that has been shown to work well for patients and clinicians. Some streamlining to achieve a balance between rigour and practicality will be essential if the anticipated demand for PtDAs is to be met.

We would urge PtDA developers to look further afield for examples of development processes and quality guidelines that may be instructive, for example guidance on developing clear communications or user-centred web design [26,27]. Different formats and delivery mechanisms also require more evaluation, for example web-based formats with electronic links to clinical record systems. It makes little sense to embark on the development of a PtDA without considering the context in which it will be used and how it will be made available to patients and clinicians. Better understanding of the barriers and facilitators to adoption of shared decision making and the needs of the various stakeholders will be essential to ensure successful development and implementation of high quality, useful, and relevant decision aids.

Most PtDA developers aim to produce reliable, unbiased, clear and comprehensible representations of clinical options and outcomes that are tailored to patients’ needs and fit into clinicians’ work flows in a feasible manner, but their achievement of these goals cannot simply be taken on trust. Users adopting PtDAs in practice look for evidence of careful user-testing and clear, transparent documentation. We have included a suggested template for reporting key elements in the development process (see Table 1). Developers might choose to make this information publicly available on their websites or by other means, or they may prefer to seek accreditation from a third party. There are now efforts under way in several countries to develop systems for certifying patient decision aids as part of regulations supporting widespread use of certified PtDAs in routine practice. Any accreditation scheme will require a set of agreed-upon standards and careful documentation of the processes by which the PtDA was developed. The IPDAS criteria could form the basis of such schemes. We hope our suggested model of the development process will help those wishing to raise standards in decision aid development.

**Table 1:** Template for Documenting PtDA Development Process

1.	Scope a. Described health condition or problem b. Stated the decision that needs to be considered c. Specified target audience. d. Explicitly identified guiding theoretical framework, if applicable
**2.**	**Steering Group** a. Included patients, clinicians, other experts (patient educators, shared decision making expertise, policy makers, others) b. Membership clearly identified including credentials c. Conflict of interest identified, if applicable

**3.**	**Design** a. Elicited patients’ views on patients’ information and decision support needs (reported method) b. Elicited clinicians’ views on patients’ information and decision support needs (reported method) c. Described format (media and format) with rationale d. Described intended setting e. Explicitly described timing of introduction into patient pathway, how and when decision aid will be distributed to patients and/or clinicians) f. Appraised and summarized quality of clinical evidence relevant to the decision and options, described methods for evidence review g. Described prototype development

**4.**	**Alpha testing** (comprehensibility and usability) a. Reviewed by patients / family members b. Reviewed by clinicians c. Reviewed by other experts (specify: ____)

**5.**	**Beta testing** in “real world setting” (feasibility) a. Data collected on patients’ experience of using PtDA b. Data collected on clinicians’ experience of using PtDA c. Peer review by experts external to development process

## Competing interests

Angela Coulter (AC) provides part-time paid consultancy and Diana Stilwell (DS) receives salary support as Chief Production Officer for the Informed Medical Decisions Foundation, a not-for-profit organisation that develops content for patient decision aids. The Foundation has an arrangement with a for-profit company, Health Dialog, to co-produce these programmes. The programmes are used as part of the decision support and disease management services that Health Dialog provides to patients.

Jennifer Kryworuchko (JK), Patricia Mullen (PM), Chirk-Jenn Ng (CN), and Trudy van der Weijden (TW) have no competing interests to declare.

## Authors’ contributions

AC led the research, participated in the study design and article review, carried out the analysis and drafted the manuscript with DS; all other authors participated in the study design and article review and were involved in revising the manuscript. All authors have given final approval of the version to be published.

## Supplementary Material

Additional File 1 **Appendix 1:** Review Checklist Click here for file

Additional File 2 **Table S1:** Key Elements of Decision Aid Development Process Click here for file

## Declarations

Publication of this supplement was partially funded by an unrestricted grant from the Informed Medical Decisions Foundation. The remaining costs were funded by the authors' departmental resources. Administrative and editorial support for this supplement was also provided by The University of Texas MD Anderson Cancer Center.

This article has been published as part of *BMC Medical Informatics and Decision Making* Volume 13 Supplement 2, 2013: The International Patient Decision Aid Standards (IPDAS) Collaboration’s Quality Dimensions: Theoretical Rationales, Current Evidence, and Emerging Issues. The full contents of the supplement are available online at http://www.biomedcentral.com/bmcmedinformdecismak/supplements/13/S2.
