# Supplementary material for: A systematic development process for patient decision aids
Source: BMC Med Inform Decis Mak. 2013 Nov 29;13(Suppl 2):S2. doi: 10.1186/1472-6947-13-S2-S2 (PMC4044159; doi:10.1186/1472-6947-13-S2-S2)
Supplement: Additional File 1 — Appendix 1: Review Checklist [file 1472-6947-13-S2-S2-S1.pdf]

Appendix 1: Review Checklist

IPDAS update: Using a systematic development process

-- Framework for review --

1. Starting point: key aspects of systematic development process not covered by other IPDAS groups (see below)
2. Include RCTs selected for next update of Cochrane Collaboration review (86 trials, 105 papers)
3. Assess description of development process in main paper and/or any subsidiary papers
4. Make qualitative judgment on validity of the process.

**Main study** (i.e., paper that describes main results of RCT of decision aid)<br>*PLEASE INSERT REFERENCE HERE:*	
**DESCRIBES SCOPE AND PURPOSE**	
[1a-c] Does paper describe scope and purpose of the decision aid? (i.e., describes the health condition or problem; states the decision(s) that need to be considered; specifies the target audience) – yes/no/cites another paper (give ref)	[1a] Does paper describes the health condition or problem?<br>□ YES<br>□ NO<br>□ Cites another paper → Specify:<br>[1b] Does paper state the decision(s) that need to be considered?<br>□ YES<br>□ NO<br>□ Cites another paper → Specify:<br>[1c] Does paper specify the target audience?<br>□ YES<br>□ NO<br>□ Cites another paper → Specify:
[1d] What is described in main or subsidiary paper in relation to scope and purpose?	
[1e] Any comments on the validity of the description of scope and purpose?	

DESCRIBES THEORETICAL FRAMEWORK	
[2a] Does paper make reference to a theory or theoretical framework that informed the development of the decision aid? – yes/no/cites another paper (give ref)	□ YES<br>□ NO<br>□ Cites another paper → Specify:
[2b] What is described in main or subsidiary paper in relation to the theoretical framework, i.e. what are its theoretical underpinnings?	
[2c] Any comments on the validity of the description of theoretical framework?	
**DESCRIBES METHODS FOR ASSESSING DECISIONAL NEEDS**	
[3a-b] Does paper describe how patients' decisional needs were assessed? (i.e. what methods were used to consult patients; how many patients were consulted; results of patient consultation) – yes/no/cites another paper (give ref)	[3a] Does paper describe what methods were used to consult patients?<br>□ YES<br>□ NO<br>□ Cites another paper → Specify:<br><br>[3b] Does paper describe how many patients were consulted?<br>□ YES<br>□ NO<br>□ Cites another paper → Specify:<br><br>[3c] Does paper describe results of patient consultation?<br>□ YES<br>□ NO<br>□ Cites another paper → Specify:

[3d] What is described in main or subsidiary paper in relation to patients' decisional needs?	What type of decision aid was this?<br><br>□ TREATMENT<br>□ SCREENING<br><br>What proportion of patients wanted information about:<br><br><u>Treatment</u> / <u>Screening</u><br><br>health condition? % / health condition? %<br>treatment options? % / 'no test' option? %<br>'no treatment' option? % / test procedures %<br>treatment procedures % / risks of procedures %<br>potential benefits % / rates of true/false positives/negatives %<br>potential risks % / consequences of positive result %<br>consequences of negative result %
[3e] Any comments on the validity of the assessment of patients' decisional needs?	

[3f] Does paper describe a method for finding out what professionals need to prepare them to discuss a specific decision with patients? – yes/no/cites another paper (give ref)	◻ YES<br>◻ NO<br>◻ Cites another paper → Specify:
[3g] What is described in main or subsidiary paper in relation to professionals' decisional needs?	What proportion of professionals wanted information about:<br><br>Treatment / Screening<br><br>health condition? % / health condition? %<br>treatment options? % / 'no test' option? %<br>'no treatment' option? % / test procedures %<br>treatment procedures % / risks of procedures %<br>potential benefits % / rates of true/false positives/negatives %<br>potential risks % / consequences of positive result %<br>consequences of negative result %
[3h] Any comments on the validity of the assessment of professionals' decisional needs?	

DESCRIBES METHODS FOR REVIEW AND FIELD TESTING	
[4a] Does paper describe how the decision aid was reviewed by external patients (i.e. those not involved in producing the decision aid)? – yes/no/cites another paper (give ref)	□ YES<br>□ NO<br>□ Cites another paper → Specify:
[4b] What is described in main or subsidiary paper in relation to external review by patients?	
[4c] Any comments on the validity of the external review by patients?	
[4d] Does paper describe how the decision aid was reviewed by external professionals (i.e. those not involved in producing the decision aid)? – yes/no/cites another paper (give ref)	□ YES<br>□ NO<br>□ Cites another paper → Specify:
[4e] What is described in main or subsidiary paper in relation to external review by professionals?	
[4f] Any comments on the validity of the external review by professionals?	

[4g] Does paper describe how the decision aid was field tested with patients who were facing the decision? – yes/no/cites another paper (give ref)	◻ YES<br>◻ NO<br>◻ Cites another paper → Specify:
[4h] What is described in main or subsidiary paper in relation to field testing with patients?	
[4i] Any comments on the validity of the field testing with patients?	
[4j] Does paper describe how the decision aid was field tested with practitioners who counsel patients who face the decision? – yes/no/cites another paper (give ref)	◻ YES<br>◻ NO<br>◻ Cites another paper → Specify:
[4k]What is described in main or subsidiary paper in relation to field testing with professionals?	
[4l] Any comments on the validity of the field testing with professionals?	

DESCRIBES DISSEMINATION AND IMPLEMENTATION PLAN	
[5a-e] Does paper describe how the decision aid will be disseminated to patients/professionals and how it will be implemented (including rationale for choice of platform)/mode of presentation; setting – primary care/secondary care/use by health coaches, etc.)? – yes/no/cites another paper (give ref)	5a Does paper describe how the decision aid will be disseminated to patients?<br>□ YES<br>□ NO<br>□ Cites another paper → Specify:<br><br>5b Does paper describe how the decision aid will be disseminated to professionals?<br>□ YES<br>□ NO<br>□ Cites another paper → Specify:<br><br>5c Does paper describe how it will be implemented (including rationale for choice of platform)?<br>□ YES<br>□ NO<br>□ Cites another paper → Specify:<br><br>5d Does paper describe mode of presentation?<br>□ YES<br>□ NO<br>□ Cites another paper → Specify:<br><br>5e Does paper describe setting?<br>□ YES, Primary Care setting<br>□ YES, Secondary Care setting<br>□ YES, Health Coaches

	□ YES, Other → Specify:<br>□ NO<br>□ Cites another paper → Specify:
[5f] What is described in main or subsidiary paper in relation to dissemination and implementation?	
[5g] Any comments on the validity of the dissemination and implementation plans?	
**AVAILABILITY OF DOCUMENTATION ON THE DEVELOPMENT PROCESS**	
[6a- m] Does the paper refer to the availability of detailed documentation on the details of the development process (e.g. literature review methods; methods for assessing decisional needs; message/storyboard/script development; video/booklet/program development; field testing; funding source; names and credentials of developers; date of development; date of review; readability testing; evaluation; etc.)? – yes-published paper (give ref)/ yes-unpublished document/ no	[6a] Does paper refer to the availability of detailed documentation on the literature review methods?<br>□ YES, Published Paper → Specify:<br>□ YES, Unpublished document<br>□ NO<br><br>[6b] Does paper refer to the availability of detailed documentation on the methods for assessing decisional needs?<br>□ YES, Published Paper → Specify:<br>□ YES, Unpublished document<br>□ NO<br><br>[6c] Does paper refer to the availability of detailed documentation on the message/storyboard/script development?<br>□ YES, Published Paper → Specify:<br>□ YES, Unpublished document<br>□ NO<br><br>[6d] Does paper refer to the availability of detailed documentation on the

	video/booklet/program development?<br>□ YES, Published Paper → Specify:<br>□ YES, Unpublished document<br>□ NO<br><br>[6e] Does paper refer to the availability of detailed documentation on the review process?<br>□ YES, Published Paper → Specify:<br>□ YES, Unpublished document<br>□ NO<br><br>[6f] Does paper refer to the availability of detailed documentation on field testing?<br>□ YES, Published Paper → Specify:<br>□ YES, Unpublished document<br>□ NO<br><br>[6g] Does paper refer to the availability of detailed documentation on funding source?<br>□ YES, Published Paper → Specify:<br>□ YES, Unpublished document<br>□ NO<br><br>[6h] Does paper refer to the availability of detailed documentation on names and credentials of developers?<br>□ YES, Published Paper → Specify:<br>□ YES, Unpublished document<br>□ NO<br><br>[6i] Does paper refer to the availability of detailed documentation on date of development?

	☐ YES, Published Paper → Specify:<br>☐ YES, Unpublished document<br>☐ NO<br><br>[6j] Does paper refer to the availability of detailed documentation on date of review?<br>☐ YES, Published Paper → Specify:<br>☐ YES, Unpublished document<br>☐ NO<br><br>[6k] Does paper refer to the availability of detailed documentation on readability testing?<br>☐ YES, Published Paper → Specify:<br>☐ YES, Unpublished document<br>☐ NO<br><br>[6l] Does paper refer to the availability of detailed documentation on evaluation?<br>☐ YES, Published Paper → Specify:<br>☐ YES, Unpublished document<br>☐ NO<br><br>[6m] Does paper refer to the availability of detailed documentation on other aspects of development process? IF YES, → Specify Other Aspects:<br>☐ YES, Published Paper → Specify:<br>☐ YES, Unpublished document<br>☐ NO
[6n] Is there a description of what is covered in the documentation? If yes,	

please give brief outline.	
