# Supplementary material for: A systematic development process for patient decision aids
Source: BMC Med Inform Decis Mak. 2013 Nov 29;13(Suppl 2):S2. doi: 10.1186/1472-6947-13-S2-S2 (PMC4044159; doi:10.1186/1472-6947-13-S2-S2)
Supplement: Additional File 2 — Table S1: Key Elements of Decision Aid Development Process [file 1472-6947-13-S2-S2-S2.pdf]

Table S1: Key Elements of Decision Aid Development Process

Legend:
Collated from a review of papers reporting trials that were included in the latest Cochrane Collaboration's review of decision aids [1] plus associated papers describing 84 decision aids evaluated in randomized controlled trials (RCTs).

Element	Definition	Methods	Comments
Scoping	Describe health condition or problem; state the decision that needs to be considered; specify target audience	Developer advised by multi-disciplinary steering group, ideally involving topic experts, clinicians, and patients<br><br>Likely to be informed by specific theoretical approach which may be explicit or implicit.	Explicit statement should be included in PtDA.<br><br>All decision aid RCTs in Cochrane review included a description of the purpose and scope of the aid they were evaluating, but the theoretical framework underpinning the PtDA design was often unstated.
Steering Group	A team of stakeholders who advise on the development, evaluation, and implementation of the PtDA	Steering group members will have relevant expertise in decision making for the specific topic: patient representatives, clinicians, patient educators, shared decision making expertise, policy makers.	Members should be familiar with and/or sympathetic to the concept of SDM.
Design 1 and 2 – Assess decisional needs	Elicit patients' and clinicians' views on patients' information and decision support needs	Focus groups<br><br>Stakeholder interviews<br><br>Surveys<br><br>Systematic literature review (including qualitative and quantitative studies)<br><br>Direct observation	The literature contains few recommendations regarding optimal approaches to assessing decisional needs.<br><br>RCTs included in Cochrane review reported patients' perspective on decisional needs more frequently than clinicians' perspective (43% vs 15%).

Design 3 – Determine format and distribution plan	Includes choice of media and format of decision aid, setting, timing of introduction into patient pathway, how and when decision aid will be distributed to patients and/or clinicians	Formats may include print media, audio recordings, DVDs, videos, websites, computer programs, decision boards, face-to-face discussions, group education, and any combination of these.<br><br>Distribution methods include handing out in clinic, mailing, telephone coaching, or direct-to-patient via websites or other means.<br><br>Settings include primary care, secondary care, health coaches, community	Should be considered early in the development process.<br><br>Some RCTs report complex methods that may not be suitable for widespread use.<br><br>Less than a third (31%) of RCTs in Cochrane review included a description of how the decision aid would be distributed and used in routine clinical practice.
Design 4 – Review and synthesize evidence	Summary of clinical evidence relevant to the decision and options	Comprehensive literature search with emphasis on systematic reviews (when available).<br><br>The evidence may include empirical studies of patients' experience and/or preferences.<br><br>Use quality criteria to assess clinical practice guidelines when these are used as evidence source.<br><br>It may be more efficient to develop PtDAs alongside clinical practice guidelines, since they draw on the same evidence base.	Frameworks provide little guidance on selection of relevant outcomes, how to minimize bias, address potential financial conflicts of interest, reach consensus, or deal with poor-quality or inadequate evidence.<br><br>How clinical evidence was appraised and selected for inclusion in the decision aid was reported in only a small minority of RCTs included in Cochrane review (17%).
Prototype development	Draft decision aid, including storyboard, script, graphics, web design, video, etc.	Ranges from basic to highly sophisticated.	This aspect of the development process was rarely reported in any detail.
Alpha testing	Direct feedback from 'typical' users sought during the development	Review by key stakeholders (patients, clinicians) via focus groups, cognitive interviews, direct	Specific methods, observations, and results often not reported.

	process. This may include members of the steering group and others involved in the development process.	observation, usability, and acceptability testing.<br><br>Feedback may be sought at various stages in an iterative process.	RCTs included in Cochrane review reported patient testing more frequently than clinician testing (37% vs 21%).
Beta (field) testing	Testing with patients and clinicians external to the development process, where possible in 'real-world' settings, to assess feasibility.	Small-scale observational pilot studies often precede larger randomized controlled trials.<br><br>Review and field testing should be carried out with patients and clinicians who have not been involved in the development process. This may include formal peer review by specialists, as well as testing the acceptability of the content and format with practising clinicians and patients.<br><br>Offering clinicians the opportunity to review and comment on the materials, to consider how and when these might be inserted into clinical pathways and who should support their use by patients is essential if they are to be persuaded to recommend the PtDAs to their patients.	Field testing often focused on use of PtDAs in settings that may not reflect 'real-world' use; provider reactions were not routinely assessed.<br><br>RCTs included in Cochrane review reported results of field tests with patients more often than clinicians' reactions to decision aid (51% vs 19%).

References

1. Stacey D, Bennett CL, Barry MJ, Col NF, Eden KB, Holmes-Rovner M, Llewellyn-Thomas H, Lyddiatt A, Légaré F, Thomson R: **Decision aids for people facing health treatment or screening decisions.** *Cochrane Database Syst Rev* 2011, 10:CD001431.
